# Mutagenesis separates ATPase and thioesterase activities of the peroxisomal ABC transporter, Comatose

**DOI:** 10.1038/s41598-019-46685-9

**Published:** 2019-07-19

**Authors:** David J. Carrier, Carlo W. T. van Roermund, Theresia A. Schaedler, Hong Lin Rong, Lodewijk IJlst, Ronald J. A. Wanders, Stephen A. Baldwin, Hans R. Waterham, Frederica L. Theodoulou, Alison Baker

**Affiliations:** 1 School of Molecular and Cellular Biology, University of Leeds, Leeds, LS2 9JT UK; 2 Amsterdam UMC, University of Amsterdam, Laboratory Genetic Metabolic Diseases, Amsterdam, Gastroenterology & Metabolism, Meibergdreef 9, 1105 AZ Amsterdam, The Netherlands; 3 Plant Sciences Department, Rothamsted Research, Harpenden, AL5 2JQ UK; 4 School of Biomedical Sciences, University of Leeds, Leeds, LS2 9JT UK

**Keywords:** Membrane proteins, Enzyme mechanisms, Plant transporters

## Abstract

The peroxisomal ABC transporter, Comatose (CTS), a full length transporter from Arabidopsis has intrinsic acyl-CoA thioesterase (ACOT) activity, important for physiological function. We used molecular modelling, mutagenesis and biochemical analysis to identify amino acid residues important for ACOT activity. D863, Q864 and T867 lie within transmembrane helix 9. These residues are orientated such that they might plausibly contribute to a catalytic triad similar to type II Hotdog fold thioesterases. When expressed in *Saccharomyces cerevisiae*, mutation of these residues to alanine resulted in defective of β-oxidation. All CTS mutants were expressed and targeted to peroxisomes and retained substrate-stimulated ATPase activity. When expressed in insect cell membranes, Q864A and S810N had similar ATPase activity to wild type but greatly reduced ACOT activity, whereas the Walker A mutant K487A had greatly reduced ATPase and no ATP-dependent ACOT activity. In wild type CTS, ATPase but not ACOT was stimulated by non-cleavable C14 ether-CoA. ACOT activity was stimulated by ATP but not by non-hydrolysable AMPPNP. Thus, ACOT activity depends on functional ATPase activity but not vice versa, and these two activities can be separated by mutagenesis. Whether D863, Q864 and T867 have a catalytic role or play a more indirect role in NBD-TMD communication is discussed.

## Introduction

Peroxisomes are versatile, near-ubiquitous organelles that carry out a wide range of metabolic and developmental functions, dependent on the organism and tissue^1^. A common pathway is β-oxidation which mediates breakdown of fatty acids and is also involved in the synthesis of hormones and secondary metabolites^2,3^. Extensive genetic and biochemical evidence has shown that export of β-oxidation substrates from the cytosol into the peroxisome is mediated by ATP Binding Cassette (ABC) transporters belonging to subfamily D^4–6^. A core ABC transporter is comprised of four subunits: two transmembrane domains (TMDs), composed of multiple α-helices thought to bind and translocate substrates and two nucleotide binding domains (NBDs) that bind and hydrolyse ATP^7,8^. In the ATP-bound state, the two NBDs form a “sandwich dimer” with two, composite nucleotide binding sites^9–11^. Conformational changes in the NBDs are transmitted to the TMDs via the coupling helices, short α-helices located in cytoplasmic loops between two transmembrane segments of each TMD and embedded in a surface groove on the NBDs^12^.

The human genome encodes three peroxisomal ABC transporters, adrenoleukodystrophy protein (ALDP/ABCD1), adrenoleukodystrophy related protein (ADLR/ABCD2) and the 70 kDa peroxisome membrane protein, PMP70/ABCD3. These proteins are “half-size” ABCs, with the domain organisation TMD-NBD, which homodimerise to form functional transporters *in vivo*, although heterodimerisation may also occur^13,14^. The transporters exhibit distinct but overlapping substrate specificity for different classes of acyl-CoA esters^15–19^. Baker’s yeast, *S. cerevisiae* has a single peroxisomal ABC transporter, the Pxa1p/Pxa2p heterodimer, which is required for growth on media containing fatty acids as the sole carbon source^20,21^. In contrast, plant genomes encode full-size peroxisomal ABC transporters, in which the four domains are encoded as a single protein, commonly referred to as a fused heterodimer^5,22^. The prototypical plant peroxisomal transporter is Arabidopsis ABCD1/Comatose (CTS, also known as AtPxa1, Ped3, ACN2)^6^. Analysis of loss of function mutants has shown that CTS is required for β-oxidation of fatty acids and a broad range of aromatic compounds, including plant hormones and secondary metabolites^5,23–25^.

The formation of acyl-CoA esters, catalysed by acyl-CoA synthetase enzymes, is a prerequisite for the entry of substrates into the β-oxidation spiral^26^. Acyl-CoA synthetases are present in several subcellular compartments, including cytosol and peroxisomes^27^. Therefore in theory, the activation step could occur either outside or inside the peroxisome and for many years, it was unclear whether ABCD transporters accepted free acids or CoA esters as substrates (reviewed in^6^ and^28^). In an attempt to rationalise apparently contradictory evidence, a model was proposed in which CTS accepts an acyl-CoA ester, which is cleaved at some point during the transport step, such that a free fatty acid is imported and re-esterified in the peroxisomal lumen^29^. Several experimental studies support this model. Firstly, peroxisomal acyl-CoA synthetase activity, located on the matrix side of the peroxisomal membrane is required for ABCD-dependent transport in plants and yeast^29,30^. Secondly, ^18^O-labelling of β-oxidation intermediates was used to demonstrate that an acyl-CoA hydrolysis step is an obligatory step prior to oleate β-oxidation, although the source of the acyl-CoA thioesterase (ACOT) activity was not identified^31^. Finally, expression of the plant ABCD transporter, CTS in insect cells provided evidence that ACOT activity was an intrinsic property of the transporter itself and was not supplied by a separate peroxisomal protein^30^. ACOT activity was subsequently demonstrated for purified, recombinant human ABCD proteins produced in a different heterologous system^32^.

Peroxisomes contain soluble thioesterases, which play a role in regulating flux of different substrates through the pathway by releasing CoA from β-oxidation intermediates and products^33^. ABC subfamily D transporters have no obvious primary sequence homology to thioesterases and no accessory domains, which raises the question of how the thioesterase activity is provided by the protein. Acyl-CoA thioesterases belong to two classes: type I and type II^33^. The former, which are not found in yeast, insects or plants, belong to the α/β-hydrolase superfamily and possess a catalytic triad characterized by a conserved serine residue (acting as a nucleophile) located in a GXSXG motif, a conserved histidine residue and an aspartic/glutamic acid residue. The ubiquitous Type-II thioesterases belong to the Hotdog fold family of proteins, the catalytic mechanisms of which are diverse and not fully understood^34,35^. In this study, we set out to identify amino acid residues important for ACOT activity of CTS, in order to dissect the relationship between thioesterase and ATPase in the catalytic cycle. Homology modelling identified candidate residues, the functional importance of which was investigated by site-directed mutagenesis and expression in insect and yeast cells.

## Results

### Identification of residues important for ACOT activity based on homology modelling

**Figure 1:**
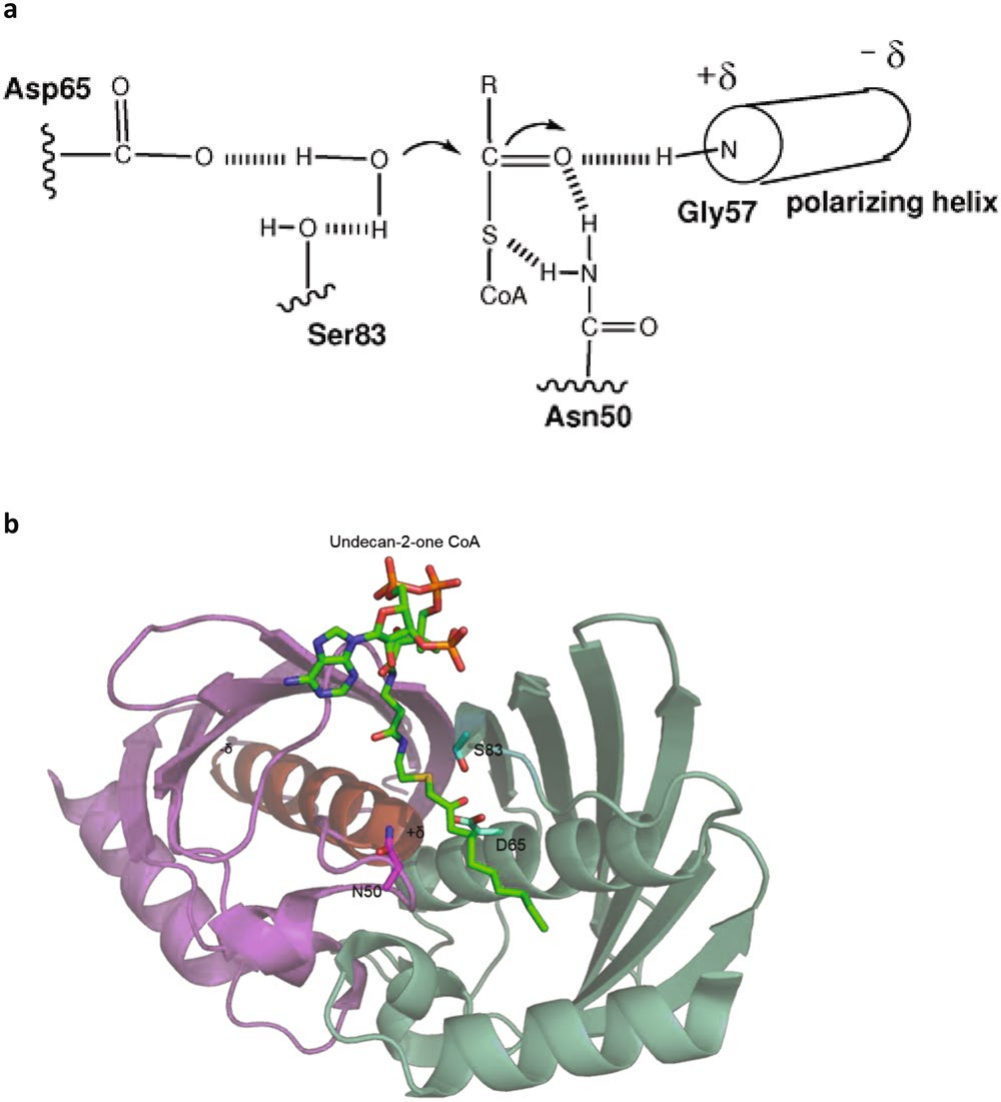
Catalytic mechanism of hotdog fold thioesterases. (**a**) Acyl-CoA thioesterase catalytic mechanism based on THEM2, reproduced with permission from^36^. (**b**) Structure of THEM2 with bound undecanon-2-one CoA, an inert substrate analogue, rendered in Pymol from 3F5O.pdb. The three catalytic residues, S83, N50 and D65 are shown as sticks. The two subunitsare indicated in green and magenta and the polarising helix is coloured red.

We hypothesised that a thioesterase catalytic site could have evolved in the context of the tertiary structure of the core transporter and based our search for residues that might contribute to the ACOT activity of ABCD transporters on the prediction that the catalytic mechanism resembles that of known Type-II thioesterases^33^. A catalytic triad has been proposed for the Hotdog-fold human thioesterase THEM2, based on biochemical evidence and a crystal structure in which an inert substrate analogue, undecan-2-one-CoA is bound in the active site (^36^, Fig. 1). Here, the aspartate (D65) and serine (S83) residues (in one subunit of the oligomeric enzyme) collaborate in the orientation and activation of the water nucleophile while the asparagine (N50), together with the N-terminal residue (glycine) of the so-called polarising helix, both in another subunit, are involved in binding and polarising the thioester carbonyl. The Asn 50 may also facilitate the departure of the thiolate leaving group (Fig. 1a). In the crystal structure (3F5O), shown in Fig. 1b, the distance between D65 and S83 is ~4 Å and between D65 and N50 ~7.5 Å. In other type-II thioesterases additional residue types, including threonine, cysteine, glutamate, glutamine and histidine have been proposed to participate in catalysis, with peroxisomal thioesterases using DQT as a catalytic triad^34,35^. Thus, it is difficult to judge which residues may be critical in the ABCD transporters, although a catalytic aspartate or glutamate seems likely to be of importance. However, we hypothesised that the residues will be conserved, located fairly close to one another, and exposed on a hydrophilic surface of the protein near the substrate-binding site^28^, which we assumed would reside in the TMDs, based on the liganded structures of other ABC transporters^37–39^.

**Figure 2:**
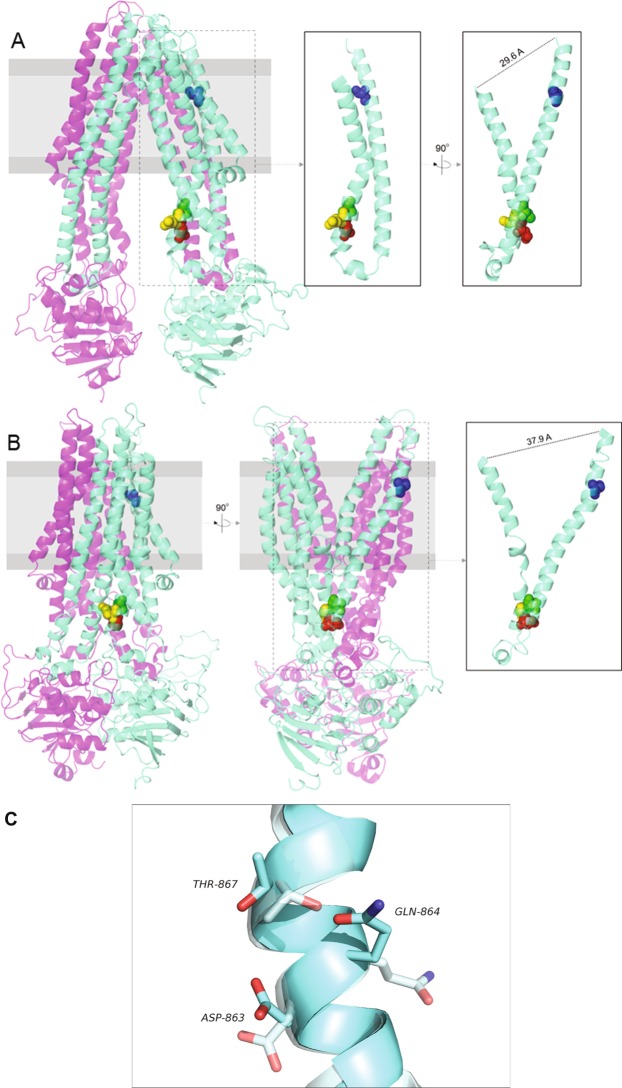
Molecular models of Arabidopsis CTS showing residue locations in both putative inward and outward conformations. Models of CTS are based on the structures of (**A**) ABCB10 in the nucleotide-free inward-facing conformation (3ZDQ.pdb), and (**B**) Sav1866 open-outward ADP-bound complex (2HYD.pdb). The The spatial relationship of the residues D863 (red), Q864 (yellow) and T867 (green), in transmembrane helix 9, to residue S810 (blue), in transmembrane helix 8, is highlighted for clarity in the side panels. (**C**) Close up of helix 9 with the inward apo confirmation in pale cyan with increased transparency and the outward nucleotide bound conformation is in normal cyan with no transparency.

To identify residues in CTS that could potentially contribute to catalysis, we employed a homology model^40^ based on the outward-facing structure (2HYD) of the bacterial ABC transporter SAV1866^12^, and also constructed a new model based on the inward-facing structure (3ZDQ) of the mitochondrial ABC transporter ABCB10 (^41^, Fig. 2). The TMDs of the models exhibit surface bands largely devoid of hydrophilic residues, compatible with an orientation facing the hydrophobic core of the bilayer, whereas the interior of this region shows a hydrophilic cavity lined with charged residues that may be involved in substrate binding and/or catalysis^28^. By analogy with the known Type-II thioesterases, we searched for conserved aspartate or glutamate residues which in the ABCB10-based model of CTS had serine, threonine or histidine residues within approximately 6 Å. CTS residue D863 was the only very highly conserved aspartate which also had a very highly conserved threonine close by (Fig. 2; Supplementary Fig S1). The next step was to look for surface-exposed, conserved glutamine and asparagine residues within about 20 Å of D863, and only one residue, Q864 met these criteria. All three residues thus identified are predicted to be located in the cytoplasmic extension of TM9, i.e. they lie outside the likely boundary of the membrane, although they may be in the head group region of the phospholipids (Fig. 2). Unlike the THEM2 structure where the catalytic residues are contributed by two different subunits, all 3 residues are on the same helix in CTS. However, their orientation changes between the two models (Fig. 2C).

### Expression and activity of CTS mutants in yeast

**Figure 3:**
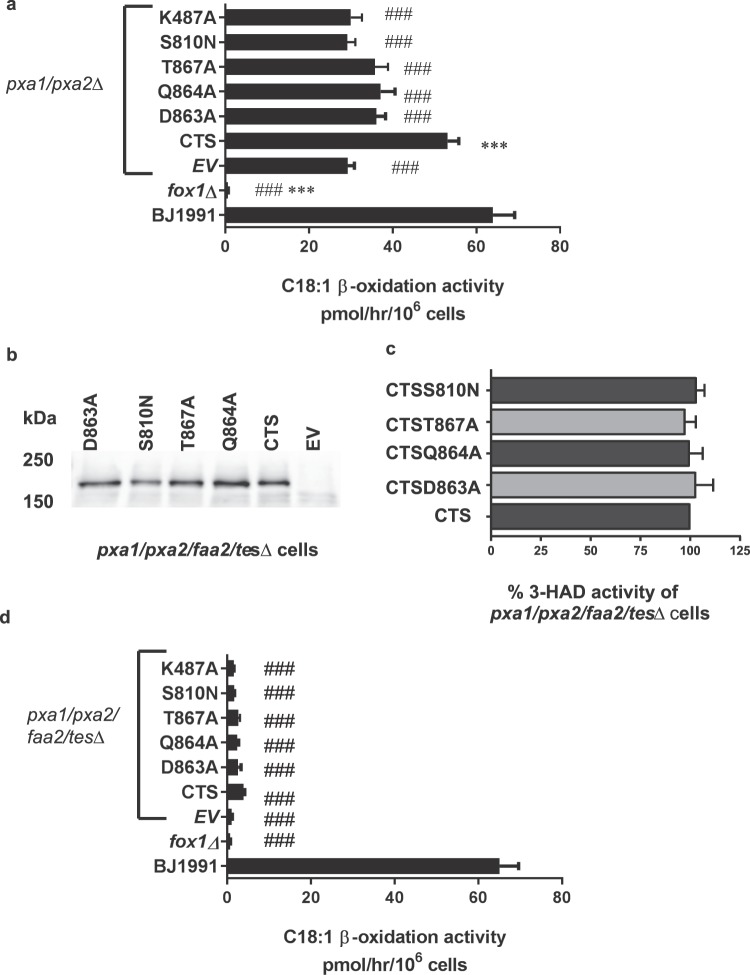
C18:1 β-oxidation in intact yeast cells. (**a**) Oleate acid β-oxidation activity in oleate-induced yeast wild-type (BJ1991) and mutant cells. The strains shown are as follows: BJ1991, *fox1Δ*, *pxa1/pxa2Δ* transformed with empty vector (EV) or expressing with CTS, D863A, Q864A, T867A, S810N, K487A. Data are represented as arithmetic means ± SD of three independent experiments. Each experiment is measured in duplicate. Asterisks indicate statistical differences (one way ANOVA, Tukeys multiple comparison test) to the *pxa1/pxa2∆*cells with *** P < 0.001 as very significant and *P < 0.05 as significant. Hash tags indicate statistical differences to BJ1991 cells with ^###^P < 0.001 as very significant and ^#^P < 0.05 as significant. (**b**) Western blot showing expression of WT and CTS mutants in *pxa1/pxa2/faa2/tesΔ* cells. EV = empty vector. (**c**) Ratio of CTS expression levels (determined by densitometry of western blots in b) to 3-HAD activity in intact cells expressed as %. (**d**) Oleate acid β-oxidation activity in oleate-induced yeast wild-type (BJ1991) and mutant cells. The strains shown are as follows: BJ1991, *fox1Δ*, *pxa1/pxa2/faa2/tesΔ* transformed with empty vector (EV) or expressing with CTS, D863A, Q864A, T867A, S810N, K487A. Data are represented as arithmetic means ± SD of three independent experiments. Each experiment is measured in duplicate. Asterisks indicate statistical differences (one way ANOVA, Tukeys multiple comparison test) to the *pxa1/pxa2/faa2/tes∆* cells with *P < 0.05 as significant. Hash tags indicate statistical differences to BJ1991 cells with ^###^P < 0.001 as very significant. *fox1Δ*, which lacks acyl-CoA oxidase, the first committed step of β-oxidation, is used as a negative control.

To test the hypothesis that residues identified by modelling are important for function, D863, Q864 and T867 were mutated individually to alanine, and expressed in yeast cells for measurement of β-oxidation activity. As reported previously^30,42^, when expressed in *pxa1/pxa2Δ* cells lacking the homologous yeast transporter, Pxa1p/2p, full-length CTS complemented β-oxidation of C18:1 to ~80% of control level of the parent yeast strain BJ1991 (Fig. 3a). β-oxidation activity was absent in the *fox1Δ* mutant, which lacks activity of acyl-CoA oxidase, the first committed step of β-oxidation. Residual C18:1 β-oxidation seen in the empty vector transformed *pxa1/pxa2Δ* cells is due to activity of the ABC transporter-independent pathway, which requires the peroxisomal acyl CoA synthetase Faa2p^20^. S810N, known to be deficient in ACOT activity^30^ and K487A, a Walker A lysine mutant deficient in ATPase activity^42^ were also expressed and displayed activity similar to empty vector controls. β-oxidation activity of D863A, Q864A and T867A mutants was significantly different to wild type activity (P < 0.05) and only 10–15% above the empty vector background, which was not statistically significant, indicating their importance for CTS function (Fig. 3a).

To remove the residual background in *pxa1/pxa2Δ* cells and any endogenous peroxisomal thioesterase activity, wild type CTS and the CTS mutants were expressed in the quadruple *pxa1/pxa2/faa2/tesΔ* cells, which lack Faa2p and the soluble peroxisomal thioesterase, Tes1p^20,43^. CTS and its mutants were expressed at similar levels in these cells (Fig. 3b,c). However wild type CTS, and the mutants’ β-oxidation activity were not significantly different to *fox1* (Fig. 3d).

**Figure 4:**
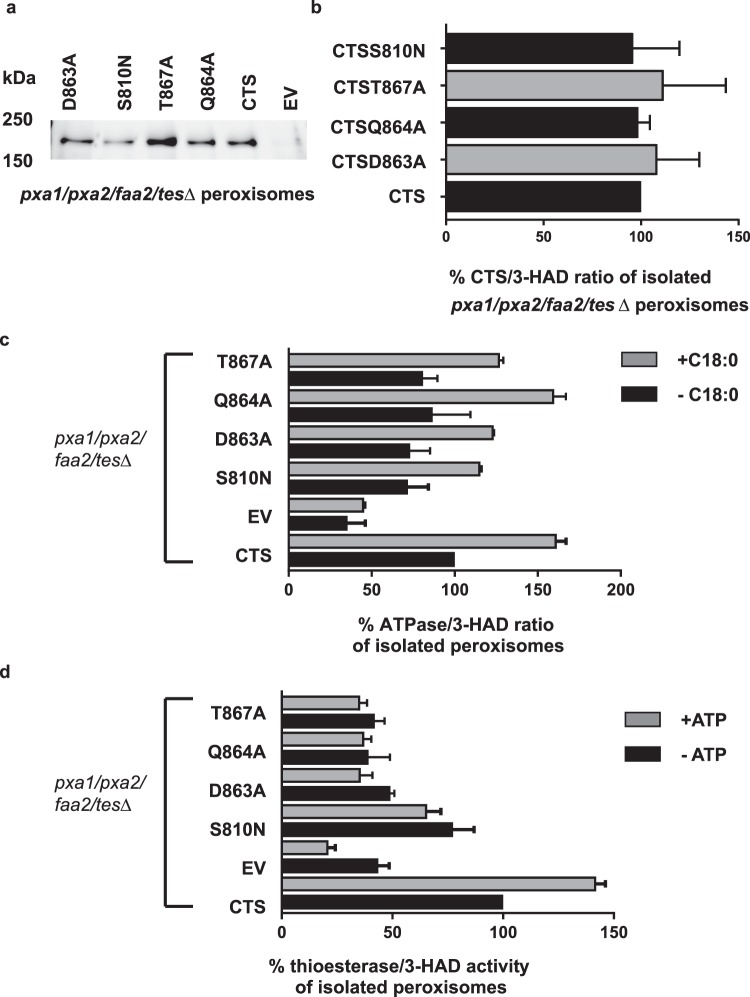
ATPase and ACOT activity in isolated yeast peroxisomes. (**a**) Western blot of yeast peroxisomes from cells expressing CTS, its mutants or empty vector (EV). (**b**) Ratio of CTS protein level (determined by densitometry of western blots in a) to 3-HAD activity. (**c**) ATPase activity and (**d**). thioesterase activity in peroxisomes isolated from *pxa1/pxa2/faa2/tesΔ* cells expressing WT CTS and mutants. Activity is expressed as a percentage of basal activity in peroxisomes from cells expressing WT CTS. An equal volume of peroxisomes was used for each assay, which was performed in duplicate and the activity normalised to the amount of peroxisomes measured by 3-HAD activity. Mean of two independent experiments ± SD.

Next, we isolated peroxisomes from the quadruple mutant lines expressing wild type CTS and mutants (Supplementary Figs S2–7. As shown in Fig. 4a, all proteins were expressed and targeted to peroxisomes. To quantify the level of expression, blots were analysed by densitometry and the values divided by the 3-HAD activity of the corresponding peroxisomal fraction. This showed that all CTS variants were present in peroxisomes at a comparable level (Fig. 4b). ATPase and ACOT activity were measured in the isolated peroxisomes and normalised to the 3-HAD activity to correct for differential recovery of intact peroxisomes (Table S2). Wild type CTS and all mutants exhibited substrate-stimulated ATPase activity of 1.4–1.6 fold (Fig. 4c). In contrast, whilst wild type CTS showed ATP-stimulated ACOT activity, the mutants did not (Fig. 4d). Even in wild type CTS, the ACOT activity measurable in yeast was very low (~1% of the ATPase activity).

### Expression and activity of CTS mutants in insect cells

**Figure 5:**
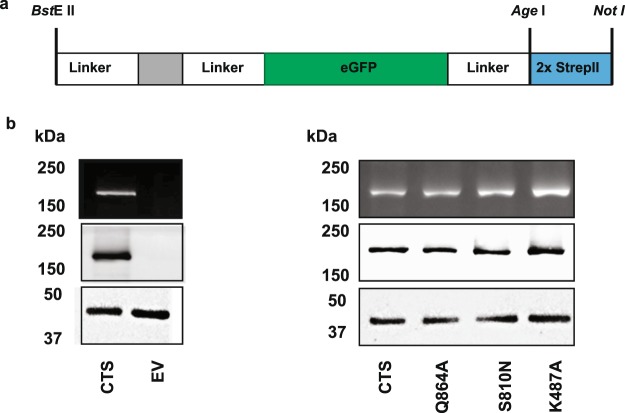
Expression of wild type and mutant CTS in insect cells. (**a**) Schematic of the C-terminal tag used in the insect cell expression construct. (**b**) Membranes (10 µg of protein) isolated from *sf9* cells, infected with empty vector (EV) or CTS baculovirus, were separated by SDS-PAGE. After measurement of the GFP fluorescence, the protein was then transferred to nitrocellulose and probed for with anti-GFP and anti-β-actin antibodies, to serve as a loading control.

**Figure 6:**
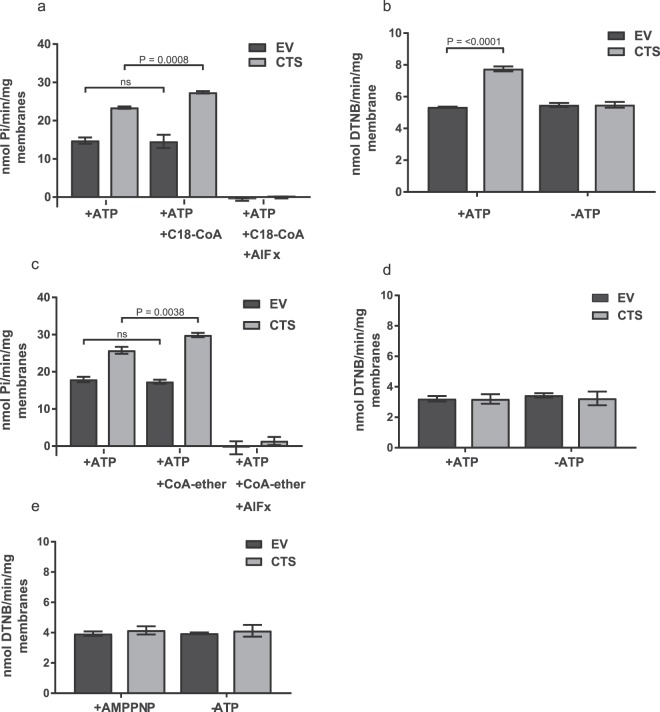
ATPase and ACOT activities of wild type CTS. The ATPase and thioesterase activities of membranes isolated from *sf9* cells infected with wild type or mutant CTS baculovirus or empty vector (EV) were measured in presence or absence of substrates and inhibitors. (**a**) ATPase, and (**b**) thioesterase activity in CTS and empty vector (EV) control membranes, in the presence or absence of 20 µM C18:0-CoA. AlFx or ATP were included as indicated. (**c,d**) as (**a,b**), respectively, but with 20 μM C14-ether-CoA. (**e**) Thioesterase activities towards 20 µM C18:0-CoA in the presence and absence of non-hydrolysable ATP analogue AMPPNP. Data were corrected for expression levels against WT CTS (using GFP fluorescence for the mutants and β-actin for EV). Values represent means ± SD from three separate experiments performed on different days from two different membrane preparations (**a,b**) or one membrane preparation (**c–e**). Statistical significance measured by one-way ANOVA, Tukey’s multiple comparison test (ns = no significance).

**Figure 7:**
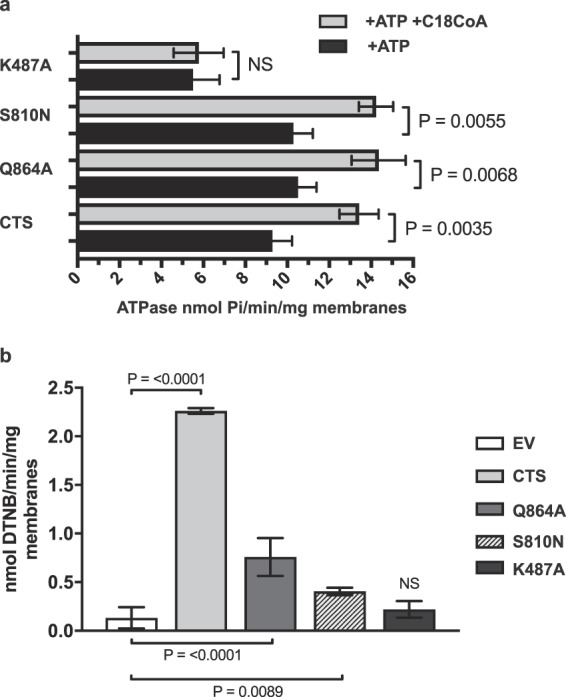
ATPase and ACOT activities in CTS mutants. (**a**) ATPase activity of wild-type and mutant forms of CTS. The background activity from empty vesicles was subtracted and the remaining activity was adjusted for protein expression level based on GFP fluorescence. The data are means ± SD of 3 three separate experiments performed on different days from a single membrane preparation. An independent membrane preparation gave qualitatively similar results. (**b**) ATP-dependent ACOT activity of wild type and CTS mutants. The difference in ACOT activity in the presence and absence of ATP for each mutant is shown. Activities were normalized for expression level of the different mutants. Values represent means ± SD from three separate experiments performed on different days from two different membrane preparations. Statistical significance measured by one-way ANOVA, Tukey’s multiple comparison test (ns = no significance).

In order to carry out more detailed biochemical analysis in a system with more robust ACOT activity, Q864A was selected as a representative mutant and was expressed together with controls S810N and K487A in insect cells^30^. Recombinant proteins were expressed as a fusion with GFP and a double Strep II tag at the C-terminus (Fig. 5a and Supplementary Fig. S8) which allowed monitoring of expression and correction of activity for protein abundance. All mutants were expressed and stable in membrane preparations from insect cells, with minor variations in expression level, as revealed by GFP fluorescence and western blotting (Fig. 5b). In all subsequent experiments activity was corrected for expression level (see materials and methods). Empty membrane vesicles (EV) exhibited both background ATPase and ACOT activity, but both activities were significantly higher in membranes from cells expressing wild type CTS (Fig. 6a,b). Activity was linear with respect to membrane protein up to 40 µg, and with respect to time up to 20 min (Supplementary Fig. S9). ATPase activity in membranes containing wild type CTS was stimulated by the substrate, C18:0-CoA and by the non-cleavable CoA analogue, C14-CoA ether, but background activity was not (Fig. 6a,c). The transition state inhibitor, AlFx inhibited both background activity and CTS-dependent activity. Background ACOT activity towards C18:0-CoA was not stimulated by ATP but ACOT activity in membranes containing CTS was stimulated approximately 1.5-fold (Fig. 6b). No ACOT activity was detected towards C14:0 ether-CoA (Fig. 6d). It was not possible to measure the effect of AlFx on ACOT activity due to precipitation, but the non-hydrolysable ATP analogue AMPPNP also did not stimulate ACOT activity, suggesting that ATP hydrolysis is important for ACOT activity (Fig. 6e). As expected, the K487A mutant has strongly reduced basal ATPase activity which was not substrate-stimulated (Fig. 7a). The other two mutants, Q864A and S810N exhibited similar ATPase activity to the wild type, and a similar degree of stimulation by C18:0-CoA, (1.45, 1.37, and 1.38, for wild type Q864A and S810N respectively) suggesting that the mutants are both competent to bind substrate (Fig. 7a). In contrast, both Q684A and S810N had severely reduced ATP-dependent ACOT activity compared to wild type CTS, as did K487A (Fig. 7b) which is consistent with the lack of ACOT stimulation by AMPPNP (Fig. 6e).

## Discussion

Remarkably, modelling enabled identification of three conserved hydrophilic residues in ABCD proteins which resembles the catalytic triad of soluble Type II thioesterases (CTS: D863/Q864/T867). However, it is important to recognise that the models were created using quite divergent template structures and the sequence identity is low in the TMDs, so they may be incorrect in detail. We tested the importance of these residues for ACOT activity by expressing mutant variants of CTS in insect cells and yeast. The mutants were expressed and stable in insect cells and in yeast peroxisomes, similar to our observations for CTS mutants *in planta*^40^ and in fibroblasts^44^. All three mutations strongly reduced β-oxidation of C18:1 in the *S. cerevisiae pxa1 pxa2Δ* background (Fig. 3), and impacted ACOT activity but retained substrate-stimulated ATPase activity (Fig. 4). In insect cell membranes, ACOT activity was strongly reduced in Q864A, but this mutant retained substrate-stimulated ATPase activity comparable to wild type CTS, strongly suggesting that this mutant protein is correctly folded and binds acyl-CoA substrate. In contrast, our data showed that the Walker A mutant, K487A has greatly reduced basal ATPase^42^ which is not substrate stimulated, and no ATP-dependent ACOT activity, despite being expressed at levels greater than wild type CTS (Fig. 7). From these data, we conclude that ACOT activity is dependent on ATPase activity but not vice versa. Consistent with this, we found that a non-cleavable CoA analogue (C14 ether-CoA) could stimulate ATPase activity of CTS (Fig. 6) in agreement with data from yeast^42^. Conversely, the non-hydrolysable ATP analogue AMPPNP did not support ACOT activity in the wild type CTS protein (Fig. 6). This could be interpreted as indicating that ATP hydrolysis is required for ACOT activity and subsequent release of substrate. It was recently shown that ATP binding causes the transition of MRP1 from the inward to outward facing conformation allowing release of substrate^45^, but whether this is universally true for all ABC transporters remains to be established. Alternatively, the sensitivity of the ACOT assay does not allow detection of activity in a single turnover event. Mutation of residue S810 to Asn had a similar effect to Q864A, resulting in retention of ATPase activity but strong reduction of ATP-dependent ACOT activity *in vitro* and impairment of fatty acid β-oxidation in yeast and plants (this work^25,30,40^). It is evident from the alignment in Supplementary Fig. S1 that S810 is not conserved in ABCD proteins (although a small side chain residue consistently occupies this position) and is therefore highly unlikely to play a catalytic role. Moreover, our homology models predict that S810 is located in TM8 which packs against TM 5 in the inward-facing conformation. Amino acid substitutions in this location may interfere with helix movements during the transport cycle.

Based on the homology model, the D/Q/T trio of residues is predicted to be located in the cytosolic extension of TM helix 9 in CTS (Fig. 2). The substrate binding site of CTS is not known, but insight is available from the structures of the glutathione-bound yeast mitochondrial ABC transporter Atm1^37^ and of its bacterial homologue NaAtm1 with bound GSSG^38^. In these structures, transmembrane helices (TMs) 4, 5 and 6 and TMs 3, 5 and 6 respectively contribute to the substrate-binding sites. Moreover, the recent structure of MRP1 reveals a binding site between the two TMDs, about 10 Å into the membrane from the cytosol, which can accommodate amphipathic substrates^39^. The substrate binding sites of these three ABC transporters all lie within the TM bundle, towards the NBD side of the membrane, thus it is unlikely that D863, Q864 and T867 are directly involved in substrate binding. Indeed, the mutants must bind substrate, since they exhibit substrate-stimulated ATPase activity (Figs 4; 6). In the absence of a structure, it is not possible to conclude whether or not the residues are involved in catalysis, especially as the homology model may not be accurate in the TMD regions. However, it is clear from our biochemical data that the three residues (D863/Q864/T867) are important for activity, consistent with their conservation and also their proximity to the transmission interface between the TMD and NBD. A possible role for these residues could be to communicate conformational change from the NBDs to stimulate ACOT activity. However, the communication is not reciprocal, since ACOT mutants can hydrolyse ATP.

CTS and Pxa1/2p are asymmetric ABC transporters, with only one consensus ATPase site in the NBD sandwich dimer^6,40^, a feature shared with several ABC transporters from other subfamilies, including MRP, CFTR and TAP1/2^46^. CTS and Pxa1/2p also exhibit asymmetry in the TMDs and the D/Q/T trio of residues identified in the model is only present in the C-terminal half of the protein. Interestingly, other ABCD proteins such as ALDP/ABCD1 are active as homodimers and thus contain two copies of the D/Q/T motif important for ACOT activity and two consensus ATPase sites. Mis-sense mutations affecting the residues corresponding to D863, Q854 and T867 have been documented in X-linked adrenoleukodystrophy patients, indicating their functional importance in symmetrical ABCD transporters (Table 1)^47^. Furthermore, an independent study demonstrated ACOT activity in human ABCD1-4 purified from *Pichia pastoris* and reconstituted into proteoliposomes^32^, demonstrating that ACOT activity is present in both symmetrical and asymmetrical ABCD proteins.

**Table 1:** X-ALD mutants corresponding to residues mutated in CTS.

Mutation in CTS (protein)	Mutation in ABCD1 (DNA)	Mutation (protein)	Occurrence	Effect on ALDP stability	Reference
D863A	c.580 G > A	D194N	2	reduced	^54^
D863A	c.580 G > C	D194H	7	60 ± 14%	^44,54,55^, unpublished
D863A	c.581_89del	D194_L197 del ins V	1	n.d.	^56^
Q864A	c.583 C > T	Q195*	1	n.d.	^47^
T867A	c.591_92insT	T198Yfs*	1	n.d.	^57^
T867A	c.593 C > A	T198K	1	n.d.	^47^
T867A	c.593 C > G	T198R	1	n.d.	^58^
T867A	c.593 C > T	T198M	2	n.d.	unpublished

The occurrence is the number of documented patients bearing the mutation; source: The ALD mutation database (http://www.adrenoleukodystrophy.info). Abbreviations: fs, frame shift; ins, insertion; n.d., not determined.

The fact that loss of ACOT activity blocks β-oxidation suggests that this is an essential step in the transport mechanism. ACOT activity could allow the acyl moiety to flip across the membrane, with the peroxisomal synthetases associated with the transporter on the trans side of the membrane^30^ effectively completing a vectorial acylation mechanism.

Our data indicate that the ATP hydrolytic cycle can still operate in the absence of ACOT activity. In insect cells expressing wild type CTS, we measure a ratio of 6:1 ATPase to ACOT activity which is in remarkably good agreement with our previous study with a slightly different wild type CTS construct (7:1)^30^ and the 5:1 ratio reported for the purified reconstituted human ABCD proteins^32^. An uncoupling of ATPase from transport has been reported for other ABC transporters, e.g. PDR5^48^ and ABCG2^49^. Intriguingly, in both these cases mutations at the transmission interface between the NBD and TMD exacerbate this effect.

Our study has predicted residues important for ACOT activity based on modelling and demonstrated their functional importance both for ACOT activity and β-oxidation. We have also demonstrated that ACOT activity depends on ATPase but not vice versa. Whilst the D/Q/T motif was identified by comparison to the catalytic triad of soluble thioesterases, these 3 residues are located close together on the same membrane helix in CTS rather than being separated on different structural elements as in thioesterases of known structure. Therefore, while it is clear they are of functional importance, whether they are components of a catalytic triad remains to be determined. The distances between them are similar in the models of the inward-facing and outward-facing conformations of CTS, although the side chain orientation is different in the inward and outward facing models; transporter movements could bring them into proximity of the bound substrate. They could alternatively be involved in communication between NBDs and TMDs which is essential for transporter rearrangements necessary for ACOT activity, rather than catalytic residues *per se*. To resolve these questions will require molecular structure of substrate bound CTS.

## Methods

### Homology modelling and analysis of residue conservation

CTS modelled on the outward-facing conformation of Sav1866 is reported in^40^. CTS was also modelled on the inward-facing structure (3ZDQ) of the mitochondrial ABC transporter ABCB10^41^, as described in^40^. Alignments of the modelled sequences with those of the templates were assisted by comparison of patterns of residue conservation and polarity within families of homologues in each case; necessary because of the great phylogenetic distances involved. Conserved residues in ABCD family transporters were identified by analysis of sequence alignments using the ConSeq approach^50^. Analyses involved alignments of 37 full-length AtABCD1 homologue sequences in plants, 214 fungal ABCD sequences and 65 metazoan ABCD sequences (excluding ABCD4 homologues). Homologues with ≥ 95% sequence identity were excluded.

### Expression of CTS in yeast cells

Mutations were introduced into plasmid pRS416/CTS (described in^42^) by site-directed mutagenesis. Primers are given in Supplementary Table S1. All constructs were sequenced to verify the presence of the mutation and to confirm that no unwanted mutations had been introduced.

### β-oxidation assay

β-oxidation assays in intact cells were performed as described previously^16^, with slight modifications. Cells were grown overnight in media containing oleate to induce β-oxidation. The β-oxidation capacity was measured in cells (OD_600_ = 2.0) in 50 mM MES, pH 6.0 supplemented with 10 μM 1-^14^C-oleate. Subsequently, ^14^C-CO_2_ was trapped with 2 M NaOH and used to quantify the rate of fatty acid oxidation in pmol/h/10^6^ cells (1OD_600_ = 1.48 × 10^7^ cells).

### Cell fractionation and isolation of peroxisomes

One liter of *S*. *cerevisiae* was grown overnight on oleate medium (YPO; OD = 1.2). Cells were harvested, washed, and incubated in 7.5 ml Buffer A (0.1 M Tris/sulfate pH 9.4) plus 10 mM DTT for 20 min at 28 °C to reduce the cell wall. Next, the cells were washed in 10 ml buffer B (1.2 M Sorbitol, 50 mM KPi pH 7.5, 1 mM EDTA) and incubated in 2 ml buffer B containing 0.75 mg/ml Zymolyase 20T. After 15–20 min, cells were centrifuged (3000 rpm 5 min), and washed with buffer C (1.2 M sorbitol, 25 mM MES pH 6.0, 1 mM KCl, 1 mM EDTA). Cells were subjected to three further rounds of resuspension in buffer D (0.6 M sorbitol, 25 mM MES pH 6.0, 1 mM KCl, 1 mM EDTA) and supernatants were combined. These combined supernatants were centrifuged at 4000 rpm for 10 min at 4 °C to remove cell debris, nuclei and large organelles. Finally, 15 ml of supernatant was centrifuged at 15,000 rpm at 4 °C in an SS34 rotor (Sorvall) for 30 min. The pellet (PP) containing peroxisomes and mitochondria were collected and gently resuspended in 1 ml of buffer D and loaded for further purification on a Nycodenz gradient (15%–20%–25%–30%–35%; w/v) in 10 mM MES-KOH, pH 6.0, 1 mM KCl, 1 mM EDTA and 8.5% (w/v) sucrose. Centrifugation was carried out in a vertical rotor at 19,000 rpm 4 °C for 150 min and fractionated in 10 fractions (fraction 1–6 1 ml; 7–10 1.5 ml). 0.83 3 ml of fraction 2, 3 and 4 were combined and diluted with 2.5 ml buffer D. After homogenization, 1.5 ml was centrifuged at 100.000 g at 4 °C. Supernatant was removed and the peroxisomal pellet (P) stored at −80 °C for further analysis. Peroxisomes were isolated in duplicate from each cell line for enzyme assays and the entire experiment was independently replicated giving a total of 4 peroxisome pellets per assay per line.

To measure the amount of peroxisomes in one P pellet, the pellet was resuspended in 200 μl PBS and the activity of the peroxisomal marker 3-hydroxyacyl-CoA dehydrogenase measured using a Cobas-Fara centrifugal analyzer by monitoring the acetoacetyl-CoA-dependent rate of NADH consumption at 340 nm^51^.

### ATPase assay in isolated yeast peroxisomes

ATPase activity was determined by a spectrophotometric assay based on enzyme-coupled reactions that utilise the ADP formed during ATP hydrolysis to oxidize NADH. Assay medium [50 mM KPi (pH 8.0), 50 mM HEPES (pH 8.0), 0.3 mM NADH, 4 mM phosphoenolpyruvate (PEP), 60 μg/ml pyruvate kinase, 32 μg/ml lactate dehydrogenase, 1 mM DTT] was pre-incubated with 15 μl isolated peroxisomes (P-pellet diluted in 200 μl PBS) for 5 min at 37 °C. The reaction was started with 25 μl of start solution (70 mM MgSO_4_, 50 mM ATP, 0.2 mM BSA plus or minus 17 μM C18:0-CoA). Final volume of the assay was 250 μl. The reaction was followed for 30 min on Cobas Fara centrifugal analyzer at 340 nm. ATPase activity was normalised to 3HAD activity.

### ACOT assay in isolated yeast peroxisomes

Acyl-CoA thioesterase activity was measured spectrophotometrically at 412 nm with 5,5′-dithiobis(2-nitrobenzoic acid) (DTNB) as described in^30^ and^52^. Isolated peroxisomes were incubated in medium containing 50 mM KPi pH 8.0, 0.2 mM DTNB, and 4 μM BSA. The reaction was started with 20 μM C18-CoA dissolved in 5% hydroxyl propylated β-cyclodextrin (HPBCD) plus or minus 10 mM ATP and followed by at 412 nm on a Cobas Fara centrifugal analyzer for 15 min at 37 °C in a final volume of 250 μl. ACOT activity was normalised to 3HAD activity.

### Expression of CTS in insect cells

To allow monitoring of expression and normalisation of CTS protein levels in insect cells, the doceca-his tag in pFASTBac1-CTS-12His^30^ was replaced with a 938 bp tandem affinity tag comprising eGFP and two copies of the Strep II tag, separated by short flexible linkers and codon-optimised for insect cells (Supplementary Fig. S8). The dodeca-his tag was excised using *Bse* EII and *Not* I and the eGFP-2xStrepII tag (synthesised by Genscript) cloned in the corresponding sites. Site-directed mutants were generated using a QuikChange II kit (Stratagene), according to manufacturer’s instructions. Primers are given in Supplementary Table S1. All constructs were sequenced to verify the presence of the mutation and to confirm that no unwanted mutations had been introduced.

### Preparation of insect membranes

All steps were varied out at 4 °C. Cells (1 l) were collected by centrifugation and resuspended in 50 ml homogenisation buffer [5 mM Tris pH 7.4, 0.5 mM MgCl_2_, 5 mM EGTA, complete protease inhibitor-EDTA (Roche), 1 mM PMSF]. DTT was added to a final concentration of 3 mM and cells were broken with 30 strokes of a tight-fitting Dounce homogeniser. The homogenate was clarified by centrifugation at 2000.g for 20 min and then membranes pelleted by centrifugation at 100,000.*g* for 1 h. Pellets were resuspended in a minimal volume of solubilisation buffer [20 mM Tris pH 7.4, 200 mM NaCl, 20% glycerol, 1 mg/ml AEBSF, complete protease inhibitor-EDTA (Roche)], snap-frozen in liquid N_2_ and stored at −80 °C.

### ATPase assays

Insect membranes (10 µg) expressing wild-type CTS or CTS mutant were mixed with buffer containing 10 mM Tris-HCl pH 7.4, 0.1 mM EGTA, 15 mM MgSO_4_, 2 mM ouabain, 10 mM sodium azide, 100 µM sodium orthovanadate, in the presence of 20 µM C18:0-CoA or ether-CoA (Avanti Polar Lipids) dissolved in 5% HPBCD, and 10 mM AlFx where indicated and incubated for 5 min at 37 °C. Reactions were started by the addition of ATP (Na_2_ATP dissolved in 7 mM MgSO_4_ pH 7.4) to give a final concentration of 5 mM and incubated 20 min at 37 °C (total volume 40 µl). The reaction was stopped by the addition of an equal volume of 12% SDS. Solutions of 1% (w/v) ammonium molybdate and 6% (w/v) ascorbate dissolved in 1 M HCl were mixed at a 1:1 ratio immediately prior to use and equal volumes added to the stopped reactions to start the colour change and incubated at room temperature for 5 min. After the addition of 120 µl of a 2% (w/v) sodium citrate, 2% (w/v) sodium metaarsenite, 2% (v/v) acetic acid solution, the plate was incubated at 37 °C for 15 min. Absorbance was read at 850 nm. ATPase activity was normalised to CTS expression level after subtraction of background from EV control.

### ACOT assays in insect cell membranes

Acyl-CoA thioesterase activity was measured spectrophotometrically using DTNB. Insect cell membranes (225 µg) were mixed at a final concentration of 0.3 mg/ml with 200 μM DTNB, 4 μM BSA, 10 mM ATP, or AMPPNP where indicated, in 50 mM potassium phosphate buffer, pH 8.0 in the presence of various concentrations of acyl-CoAs (Avanti Polar Lipids), dissolved in 5% HPBCD. The reaction was started by the addition of membranes and specific activities were determined by measuring the change in absorbance at 412 nm for 10 min. Σ = 13,800 M^−1^·cm^−1^ ^53^. Rates were corrected for expression level and non-enzymatic reduction of DTNB.

## Supplementary information

supplementary information

supplementary table 2

## Data Availability

All data generated or analysed during this study are included in this published article (and its Supplementary Information Files).
