# Supplementary material for: Mutagenesis separates ATPase and thioesterase activities of the peroxisomal ABC transporter, Comatose
Source: Sci Rep. 2019 Jul 19;9:10502. doi: 10.1038/s41598-019-46685-9 (PMC6642094; doi:10.1038/s41598-019-46685-9)

**Supplementary Material**

**Mutagenesis separates ATPase and thioesterase activities of the peroxisomal ABC transporter, Comatose.**

David J. Carrier^1,4^, Carlo W.T. van Roermund^2,4^, Theresia A. Schaedler^3^, Hong Lin Rong^1^, Lodewijk Ijlst^2^, Ronald J. Wanders^2^, Stephen A. Baldwin^5,6^, Hans R. Waterham^2^, Frederica L. Theodoulou^3^, Alison Baker^1,7^

1. School of Molecular and Cellular Biology, University of Leeds, Leeds, LS2 9JT, UK
2. Amsterdam UMC, University of Amsterdam, Laboratory Genetic Metabolic Diseases, Amsterdam, Gastroenterology & Metabolism, Meibergdreef 9, 1105 AZ Amsterdam,

The Netherlands, T: +31 20 566 3827

1. Plant Sciences Department, Rothamsted Research, Harpenden, AL5 2JQ, UK
2. These authors contributed equally
3. School of Biomedical Sciences University of Leeds, Leeds, LS2 9JT, UK
4. Deceased
5. To whom correspondence should be addressed: [a.baker@leeds.ac.uk](mailto:a.baker@leeds.ac.uk)

**Supplementary table 2 (Excel file)**

**Supplementary Figure S1. Partial alignment of peroxisomal ABC transporters**

CTS residues selected for mutagenesis are indicated above the alignment. The residue mutated in the *ped3-4* allele of CTS (S810N; [1]), which reduces thioesterase activity ([2] and this study) is highlighted in yellow. The alignment was performed as described in [3]; peptide sequences of plant ABC transporters were divided in the linker region which separates NBD1 and the first TM helix of TMD2 [4]. Residues in the alignment are highlighted to indicate the extent of conservation in an alignment of 85 homologous sequences from ABC transporter subfamily D members, of which those shown represent a subset: black, dark blue and bright blue respectively indicate residues that are identical in 40-49%, in 50-74% and in ≥ 75% of the 85 sequences. The predicted positions of TM helices (derived from the homology model) are indicated in black, and where these extend into the cytoplasm they are shown in red. The putative coupling helix 1 (CH1) is also shown. Abbreviations: *At,* *Arabidopsis thaliana*; *Os*, *Oryza sativa*; *Hv*, *Hordeum vulgare*; *Mt*, *Medicago truncatula*; *Pt*, *Populus trichocarpa*; *Hs*, *Homo sapiens*; *Sc*, *Saccharomyces cerevisiae*.

**Supplementary Figure S2. Fractionation of peroxisomes from *pxa1 pxa2 faa2 tesΔ* strain transformed with CTS.**

Cells were cultured overnight on oleate rich medium and cells were fractionated into homogenate (H) and organellar pellet (MP; a, b) which was subsequently separated on a Nycodenz gradient (c). Fumarase (pale grey bars) and 3-HAD (black bars) were assayed as markers for mitochondria and peroxisomes respectively. Fractions 2, 3 and 4 from the gradient were diluted twice, collected by centrifugation, to give a peroxisome pellet (P) and stored at minus 80°C. Resuspended pellets were assayed for 3-HAD and fumarase activity (A, B) and used for ATPase and thioesterase assays (Fig. 4).

**Supplementary Figure S3. Fractionation of peroxisomes from *pxa1 pxa2 faa2 tesΔ* strain transformed with empty vector.**

Details as for Supplementary Figure S2.

**Supplementary Figure S4. Fractionation of peroxisomes from *pxa1 pxa2 faa2 tesΔ* strain transformed with CTS_D863A_.**

Details as for Supplementary Figure S2.

**Supplementary Figure S5. Fractionation of peroxisomes from *pxa1 pxa2 faa2 tesΔ* strain transformed with CTS_Q864A_.**

Details as for Supplementary Figure S2.

**Supplementary Figure S6. Fractionation of peroxisomes from *pxa1 pxa2 faa2 tesΔ* strain transformed with CTS_T867A_.**

Details as for Supplementary Figure S2.

**Supplementary Figure S7. Fractionation of peroxisomes from *pxa1 pxa2 faa2 tesΔ* strain transformed with CTS_S810N_.**

Details as for Supplementary Figure S2.

**Supplementary Figure S8. Sequence of affinity tag for detection and quantification of CTS in insect cells.**

GFP sequence is highlighted in green, Strep II tag highlighted in red; linker regions highlighted in yellow and restriction sites in bold and underlined.

**Supplementary Figure S9 Linearity of ATPase activity with protein concentration and time.**

ATPase assays were performed on membranes isolated from *sf9* cells expressing CTS or infected with empty vector control, varying the amount of membrane protein assayed (a), time (b). Values represent mean ± SD, after subtraction of background phosphate levels, from six replicates of the same membrane preparation.

**Supplementary figure S10. Complete western blot used for Fig 3b**

**Supplementary Figure S11 Complete western blot used for Fig 4a**

**Supplementary Figure S12 Complete western blots used for figure 5b**

**Supplementary referencesCo**

1. Hayashi, M., *et al.* Ped3p is a peroxisomal ATP-binding cassette transporter that might supply substrates for fatty acid beta-oxidation. *Plant Cell Physiol*. **43**, 1-11 (2002).

2. De Marcos Lousa, C., *et al*. Intrinsic acyl-CoA thioesterase activity of a peroxisomal ATP binding cassette transporter is required for transport and metabolism of fatty acids. *Proc Natl Acad Sci U S A*. **110**, 1279-1284 (2013).

3. Dietrich, D., *et al*. Mutations in the Arabidopsis Peroxisomal ABC Transporter COMATOSE Allow Differentiation between Multiple Functions In Planta: Insights from an Allelic Series. *Mol Biol Cell*. **20**, 530-543 (2009).

4. Nyathi, Y., *et al*. Pseudo half-molecules of the ABC transporter, COMATOSE, bind Pex19 and target to peroxisomes independently but are both required for activity. *FEBS Lett.* **586**, 2280-2286 (2012).

**Table S1 Primers used in this study**

| **Primer** | **Sequence (5’-3’)** |
| --- | --- |
| S810Nfor | GAGTGTTCTCCAAAATGGTGCATCTTC |
| S810Nrev | GAAGATGCACCATTTTGGAGAACACTC |
| D863Afor | CAATAGTATTGATGCGGCCCAGAGACTCACTCGTG |
| D863Arev | CACGAGTGAGTCTCTGGGCCGCATCAATACTATTG |
| Q864Afor | GTATTGATGCGGACGCGAGACTCACTCGTG |
| Q864Arev | CACGAGTGAGTCTCGCGTCCGCATCAATAC |
| T867Afor | GATGCGGACCAGAGACTCGCTCGTGACCTGGAAAAG |
| T867Arev | CTTTTCCAGGTCACGAGCGAGTCTCTGGTCCGCATC |

**Supplementary Figure S1** **Partial alignment of peroxisomal ABC transporters**

TM3/9

CH1

TM2/8

**Supplementary Figure S2. Fractionation of peroxisomes from *pxa1 pxa2 faa2 tesΔ* strain transformed with CTS.**

**Fraction number**

**% activity**

**% activity**

**c**

**b**

**a**

**Supplementary Figure S3. Fractionation of peroxisomes from *pxa1 pxa2 faa2 tesΔ* strain transformed with empty vector.**

**a**

**Fraction number**

**% activity**

**c**

**b**

**Supplementary Figure S4. Fractionation of peroxisomes from *pxa1 pxa2 faa2 tesΔ* strain transformed with CTS_D863A_.**

**Fraction number**

**% activity**

**c**

**b**

**a**

**Supplementary Figure S5. Fractionation of peroxisomes from *pxa1 pxa2 faa2 tesΔ* strain transformed with CTS_Q864A_.**

**a**

**Fraction number**

**% activity**

**c**

**b**

**Supplementary Figure S6. Fractionation of peroxisomes from *pxa1 pxa2 faa2 tesΔ* strain transformed with CTS_T867A_.**

**a**

 **Supplementary Figure S7. Fractionation of peroxisomes from *pxa1 pxa2 faa2 tesΔ* strain transformed with CTS_S810N_.**

**% activity**

**Fraction number**

**c**

**b**

**Fraction number**

**% activity**

**c**

**b**

**a**

**Supplementary Figure S8. Sequence of affinity tag for detection and quantification of CTS in insect cells.**

**Supplementary Figure S9 Linearity of ATPase activity with protein concentration and time.**

**b**

**a**

**Supplementary figure S10. Complete western blot used for Fig 3b**

**
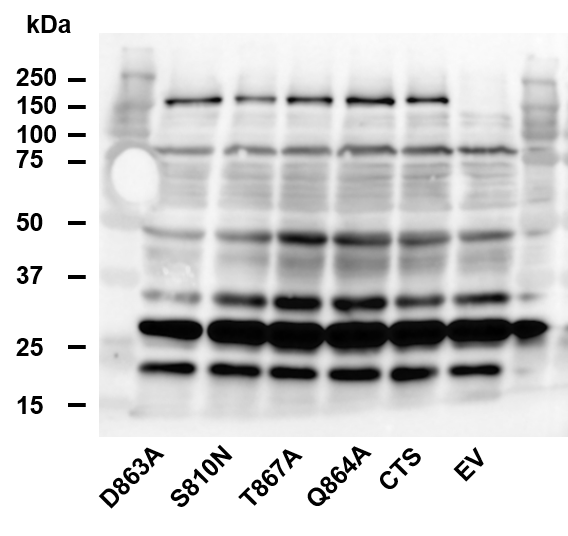
**

**Supplementary Figure S11 Complete western blot used for Fig 4a**

**
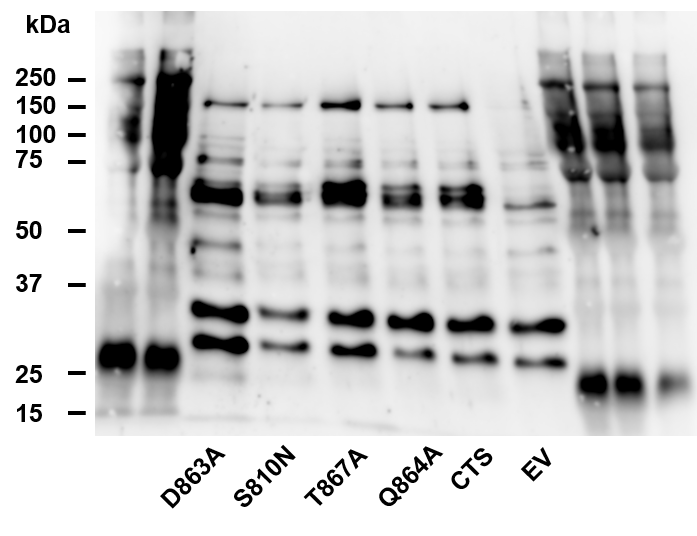
**

**Supplementary Figure S12 Complete western blots used for figure 5bGFP fluorescence,**


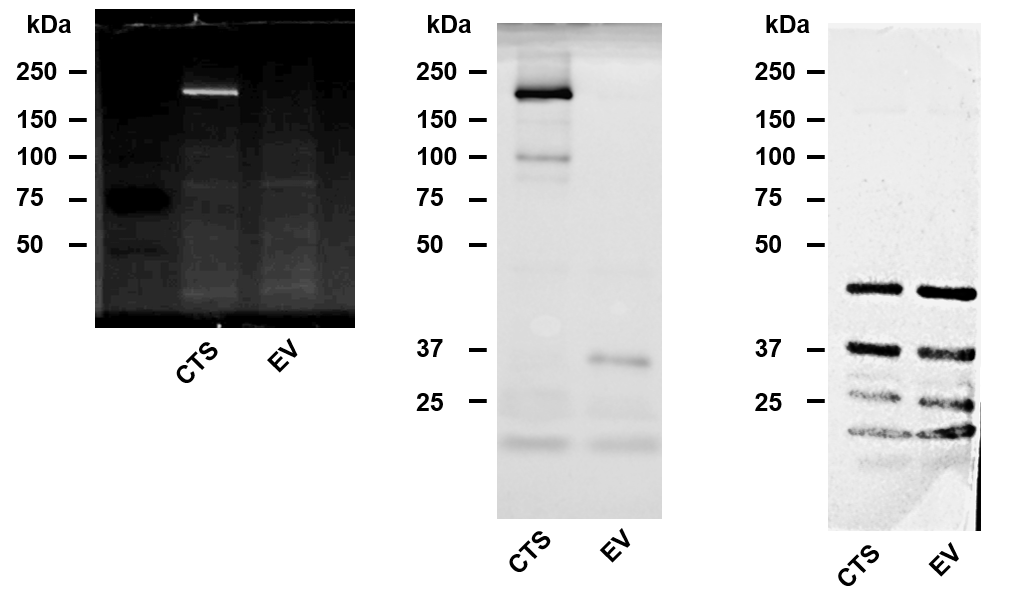
**From left GFP fluorescence, anti GFP, anti beta actin**


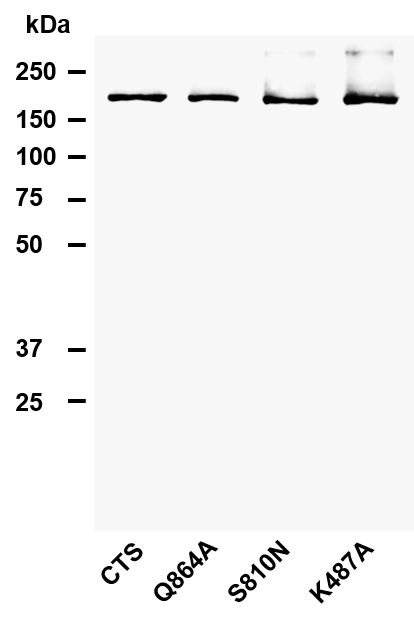

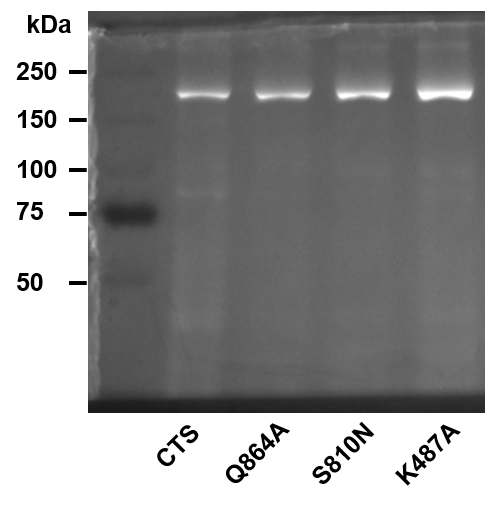
Left GFP fluorescence CTS mutants, right anti GFP

Anti beta actin CTS mutants


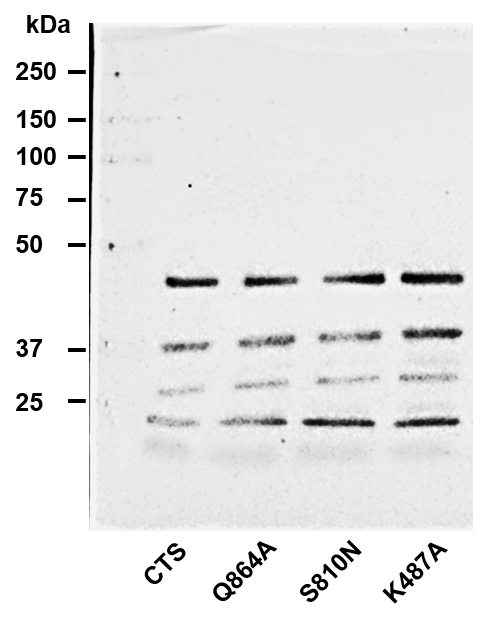

Supplement: Supplementary file 1 — supplementary information [file 41598_2019_46685_MOESM1_ESM.docx]
